# Acute Villitis and Intravascular Microorganisms in Fetal Vessels: A Case Report and Literature Review of an Unusual Histopathological Finding

**DOI:** 10.1177/1093526621993333

**Published:** 2021-02-22

**Authors:** Brenda F Narice, Martyna Trzeszcz, Marta Cohen, Dilly O Anumba

**Affiliations:** 1 Academic Unit of Reproductive and Developmental Medicine, The University of Sheffield and Sheffield Teaching Hospitals, The Jessop Wing, Sheffield, UK; 2 Department of Pathology and Clinical Cytology, University Hospital of Jan Mikulicz-Radecki, Wroclaw, Poland; 3 Histopathology Department, Sheffield Children's NHS Trust, Sheffield, UK; 4 Department of Oncology and Metabolism, The University of Sheffield and Sheffield, Sheffield, UK

**Keywords:** placental, uterine infection, villitis, sepsis

## Abstract

Optimal management of intrauterine infection to avoid serious adverse perinatal outcomes entails prompt administration of antibiotics and consideration of early delivery of the fetus to remove the focus of infection. We report an unusual case of preterm chorioamnionitis which did not improve with sensitive antibiotics, or delivery of the fetus, and ultimately required an emergency hysterectomy to save the mother’s life. Interestingly, subsequent histopathological analysis of the post-hysterectomy specimen did not reveal myometrial necrosis or infectious microorganisms. The placental pathological examination, on the other hand, showed evidence of necrotising chorioamnionitis accompanied by a rarely reported lesion: acute villitis with abundant intravascular *Escherichia coli*, a finding which is strongly associated with fetal demise and adverse maternal outcomes.

## Introduction

Acute intrauterine infection is an important contributor to adverse maternal and neonatal outcomes. Clinically, it manifests as feto-maternal tachycardia, pyrexia, uterine tenderness, maternal leucocytosis and/or foul-smelling purulent fluid or discharge from the cervical os.^1^ Suspected acute intrauterine infection can be confirmed in pregnant women by objective pre- and postnatal laboratory findings, including pathological examination of the placenta.^2^ Histologically, acute intrauterine infection comprises a: 1) maternal inflammatory response (MIR) demonstrated by the presence of neutrophilic infiltration in chorion and/or amnion in the fetal membranes and/or in the chorionic plate (acute chorioamnionitis); with or without a 2) fetal inflammatory response (FIR) demonstrated by the presence of neutrophilic infiltration in umbilical vessels with or without Wharton substance involvement and infiltration in chorionic plate vessels.^3,4^

Acute villitis, defined as the presence of neutrophilic infiltration in fetal villous capillaries and stroma, is an uncommon histological entity which may occur in isolation or accompanied by chorioamnionitis, and normally indicates severe fetal sepsis. Rarely, it may be accompanied by an abundance of microorganisms in the fetal vessels of the chorionic villi.^5,6^

In this paper, we report a rare case of severe maternal sepsis caused by necrotising preterm chorioamnionitis with acute villitis and intravascular microorganisms which only responded to hysterectomy.

## Case Report

**Figure 1.**
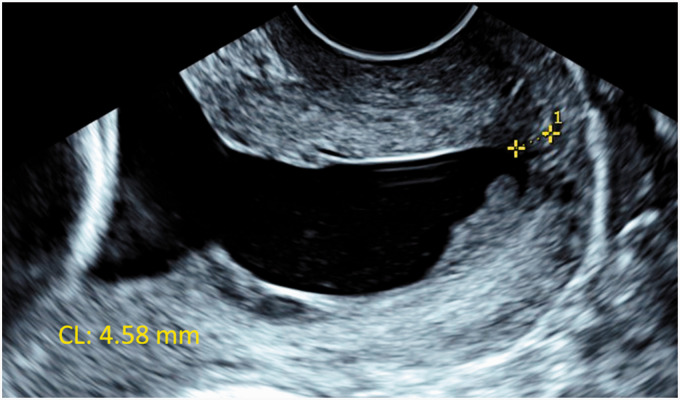
Transvaginal ultrasound of the cervix in longitudinal view at 22 weeks of gestation. The cervix was 4.58 mm long (yellow callipers), with a large funnel and intra-amniotic sludge.

A nulliparous woman of childbearing age with no pre-existing comorbidities was found to have an incidental large cervical funnel in an otherwise normal cervix (42 mm long) when she attended her regular anomaly scan at 20 weeks. Up to that point, she had not experienced any antenatal complications with normal booking bloods and low-risk results from the combined first trimester screening test for chromosomal abnormalities. A follow-up transvaginal scan two weeks later revealed the cervix had significantly shortened to 4-5 mm with a large funnel and intra-amniotic sludge, and the patient was offered a rescue cerclage (Figure 1). Unfortunately, as the suture was being secured, premature rupture of the membranes was identified and the procedure was abandoned. She was then started on a seven-day prophylactic course of erythromycin and received steroids cover for fetal lung maturation. Following completion of her antibiotics course, the patient remained clinically well with normal-range inflammatory markers for another week. Microbial high-vaginal swabs analysis did not reveal any pathogenic fungal or bacterial growth. When the metabolomic fingerprint of the cervicovaginal fluid was further analysed by Raman Spectroscopy, no evidence of altered carbohydrate metabolism normally exhibited by the subclinical overgrowth of preterm birth-associated anaerobic bacteria was identified.^7^

At 24 weeks and 4 days, the patient suddenly felt unwell, and became septic with significantly raised venous lactate levels (>6 mmol/L). Fetal demise was confirmed at this point. Despite high-flow oxygen, intravenous broad-spectrum antibiotics and aggressive fluid resuscitation, the patient continued to deteriorate and became hypotensive, tachycardic, hypoxic and pyrexial. A decision to expedite delivery was made, and a hysterotomy under general anaesthesia was performed on the same day. The placenta was sent for histopathologic examination and no delay in fixing the sample at delivery theatre was noted.

Intraoperatively, the patient remained haemodynamically unstable requiring a high dose of vasopressors and support for ventilation. The patient was then transferred to critical care for management of her sepsis. Antibiotics were escalated to meropenem and clindamycin as preliminary blood cultures, and placental and fetal swabs reported the presence of Gram negative microorganisms later confirmed to be *Escherichia coli*. By the second day post-hysterotomy, the patient continued to develop signs of multi-organ failure. An urgent chest, abdomen and pelvis computed tomography was performed to rule out a collection and failed to identify a source of sepsis. In view of the patient’s worsening condition, and on suspicion of myometrial necrosis, a hysterectomy with conservation of tubes and ovaries was performed 36 hours after delivery of the fetus. Soon after the surgery, the patient experienced a marked clinical improvement and continued to recover in the following weeks.

**Figure 2.**
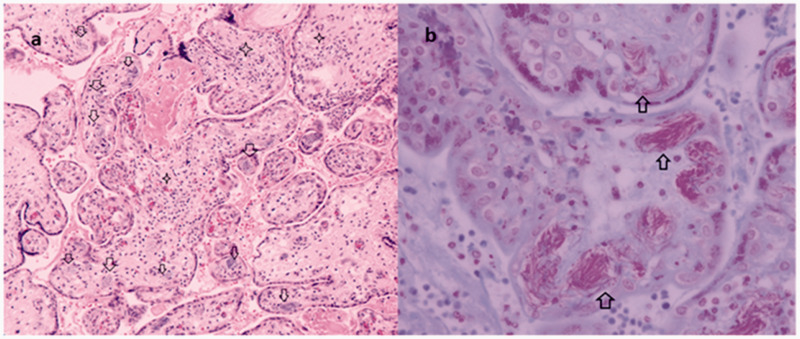
A, The microscopic evaluation of the placenta delivered at 24 weeks and 4 days of gestation complicated by a maternal septic shock requiring hysterectomy 36 hours after delivery: placental villi showing villitis (stars) associated to the presence of bacterial organisms in fetal vessels (arrows) (H&E × 20). B, Gram Twort stain shows red bacilli filling the fetal blood vessels in villi (arrows × 60). The microorganisms were located within the large number of fetal placental vessels, involving all villous subdivisions of the villous tree ramifications. Affected vessels were tightly filled with bacteria.

Thorough histological analysis of the uterus, however, did not identify any evidence of retained products of conception, myometrial necrosis or infectious microorganisms, only interstitial haemorrhage with focal and perivascular acute inflammatory cells close to the low uterine segment incision. The analysis of the placenta, on the other hand, confirmed the diagnosis of acute necrotising chorioamnionitis (MIR = stage 3, grade 2; FIR = stage 3, grade 2) associated with acute villitis and numerous fusiform Gram negative microorganisms located within the large number of fetal vessels from stem to terminal villi, which were later confirmed to be *Escherichia coli* (Figure 2). No fetal autopsy was performed as per parents’ wishes.

## Discussion and Conclusion

**Figure A1.**
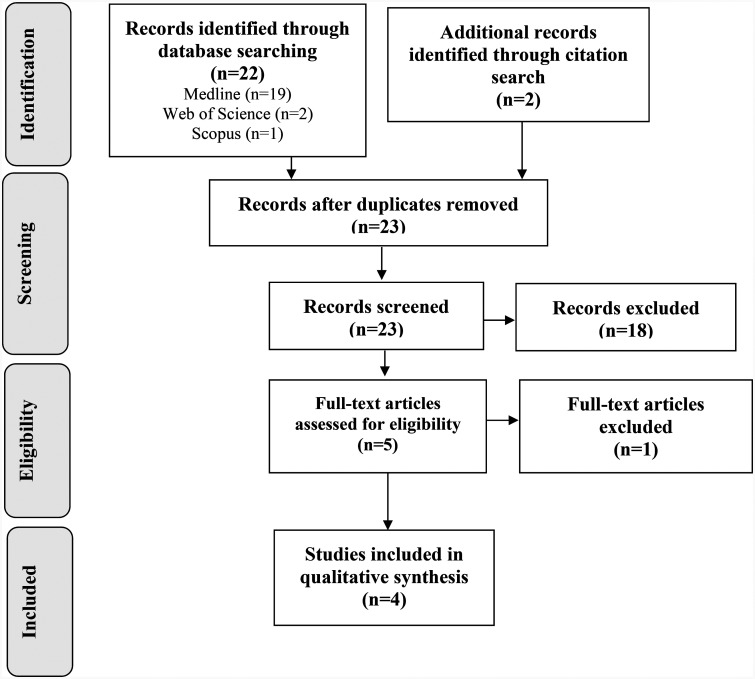
The PRISMA flow chart shows the methodology for the selection of cases reporting on bacterial organisms filling the intravascular spaces of the chorionic villi.

The presence of bacteria within the placental fetal capillaries constitutes a rarely reported histological finding.^5,6,8^ A comprehensive electronic and hand search performed in Medline via OvidSP, Scopus and Web of Science from inception to May 2020 only yielded three case reports^5^,^8^ and one short case series^6^ (Appendix 1: Figure A1).

In all the cases published, including the one reported in this paper, the presence of intravascular microorganisms in the fetal capillaries of the chorionic villi has been associated, without exception, with second-trimester fetal demise (Table 1). The analysis of the placenta provided key information about the timing of the infection and the causative agents. The presence of bacterial microorganisms in the fetal vasculature was accompanied by acute villitis, an atypical inflammatory fetal response (Table 1). Evidence of neutrophil infiltration in the fetal capillaries and chorionic villi demonstrates that the bacteria were present in fetal circulation while the fetus was still alive rather than postnatally or due to placental contamination.^4,6,9^ The degree of fetal inflammatory response seen in these placentas suggests that the fetal septicaemia was severe enough to cause fetal death. Placental analysis using appropriate immunohistochemistry and PCR testing also enabled to identify the infective agents in almost all the cases reported even when urine, blood and placenta microbiology cultures had been inconclusive.^6^ The leading species were *Escherichia coli* and Group B Streptococcus which have well-documented associations with ascending polymicrobial intrauterine infections^10^ (Table 1).

**Table 1.** Reported Cases of Placental Fetal Intravascular Microorganisms.

Study	Gestational Age	Bacteria	Clinical Outcomes		Histological Findings	
			Maternal	Fetal	Maternal	Fetal
Sheikh et al.^11^	18 weeks (n = 1)	*Klebsiella pneumoniae*	Sepsis	Death	Chorioamnionitis	Acute villitis
Matoso et al.^5^	17 weeks (n = 1)	*GBS*	Sepsis	Death	Chorioamnionitis	Acute villitis
Bae et al.^8^	21 weeks (n = 1)	*GBS*	Sepsis	Death	Chorioamnionitis	Acute villitis
Schubert et al.^6^	16-27 weeks (n = 13)	*E.coli* (n = 8) *GBS* (n = 3) *GPC* (n = 2)	Sepsis (n = 9) ICU admission (n = 2) Death (n = 1)	Death (n = 13)	Chorioamnionitis (n = 11)	Acute villitis (n = 13)
Present case	24 weeks (n = 1)	*E.coli*	Sepsis ICU admission Hysterectomy	Death	Chorioamnionitis	Acute villitis

*GBS: Group B Streptococcus, E.coli: Escherichia coli, GPC: Gram positive cocci, ICU: intensive care unit.*

Maternal sepsis secondary to chorioamnionitis is a rare occurrence even in the context of premature rupture of membranes with some studies quoting rates of less than 1%. Admission to intensive unit remains uncommon, and prognosis is generally good.^10,12^ However, when there is presence of intravascular microorganisms in the placental villi, maternal morbidity and mortality rate have been noticed to be significantly higher than any other intrauterine infection.

In over seventy-five percent of all the cases reported (n=13), women became septic; three of whom required intensive-unit support (23%), one underwent hysterectomy and one died (8%, Table 1).

The few available cases of acute villitis and intravascular microorganisms identified in the literature were reported in high-income countries (Table 1). In these settings, peripartum hysterectomies are rarely performed (approximately 0.7:1,000 pregnancies), with the leading cause being morbidly adherent placenta rather than chorioamnionitis. In our tertiary maternity unit which oversees 7,000 births per year, for example, this is one of the first times uterine, placental and clinical outcomes have been triangulated in the context of a life-threatening intrauterine infection given the rarity of performing peripartum hysterectomies for puerperal sepsis.

On the contrary, in low-income countries, feto-maternal sepsis remains a relatively usual indication for hysterectomies, which along with uterine rupture and intractable postpartum haemorrhage complicates at least 2.8 every 1,000 pregnancies.^13–15^ However, histopathological analysis of the uterus, placenta and fetus remains a relatively uncommon event in these settings which limits our understanding of the true incidence of acute villitis associated with intravascular microorganisms and leads to potential reporting bias.^16^

Traditionally, in high-income countries, the gold-standard clinical management of intrauterine infection comprises broad-spectrum antibiotics and removing the focus of infection by early delivery of the fetus.^2^ In the case we report, the patient failed to respond to conventional treatment, but greatly improved after the uterus was removed. A thorough histopathological analysis of the uterus, however, did not reveal any evidence of uterine infection. Although it remains a possibility that sampling may have missed a focus of persistent infection in the uterus, 14 extensive samples taken from the cervix, the right and left cornua, the uterine lower and anterior aspects, the fundus, and the included resection margins did not show any fungal or bacterial infection. As no samples were taken from the uterine veins at the parametrial margin of resection, septic venous thrombophlebitis could not be completely ruled out as a persistent focus of infection. However, it is unlikely to have been the cause for the patient’s acute decompensation as no signs of thrombosis were identified during the surgery which was performed jointly by three senior consultant gynaecologists and one senior obstetrician.

Based on the lack of response to conventional treatment, the patient’s clinical evolution after hysterectomy and the uterine and placental findings, we hypothesised that the uterus likely had been housing a large array of inflammatory cytokines secondary to the severe maternal immune response mounted against the feto-placental infection. Once this highly-active inflammatory milieu was removed during the hysterectomy, the patient made a significant clinical improvement.

As demonstrated by our case and supported by previous literature,^5,6,8^ acute villitis accompanied by the presence of intravascular microorganisms in fetal vessels is associated without exception with fetal demise, and it is more likely to be found in the context of intractable maternal sepsis. The correlation between histopathological findings and adverse feto-maternal outcomes supports a detailed histological analysis of the placenta in cases of severe acute intrauterine infection which do not respond to first-line management. Such analysis is likely not only to provide a better understanding of the origin, timing and aetiology of the inflammatory process, but also to inform current and subsequent obstetric management of these patients.

## Appendix 1: Search strategy for systematic review on human cases reporting on bacterial organisms filling the intravascular spaces of the chorionic villi

An electronic search in Medline via OvidSP, Scopus and Web of Science was performed from inception to May 2020 to identify any case and/or series cases written in English and Spanish which reported on the presence of bacteria in human fetal capillaries. We employed a combination of relevant MeSH, keywords and synonyms such as acute villitis, bacteria, chorioamnionitis and fetal intravascular microorganisms. The scoping search was supplemented by hand-searching of references and perusing of grey literature.

Database: **Ovid Medline**

<1946 to Present>

Search Strategy:

1. *Chorioamnionitis/ or *Pregnancy/ or *Adult/ or *Humans/ or Premature Birth/ or *Pregnancy Complications, Infectious/ or *Placenta/ or “acute villitis”.mp. or *Placenta Diseases/ (107427)
2. *Bacteria, Aerobic/ or *Gram-Negative Bacteria/ or *Bacteria/ or bacteria.mp. or *Bacteria, Anaerobic/ or *Gram-Positive Bacteria/ (500580)
3. chorioamnionitis.mp. or *Chorioamnionitis/ (4986)
4. *Fetus/ or *Fetal Diseases/ or *Placenta/ or "fetal intravascular organism".mp. or *Chorionic Villi/ (90274)
5. *Adult/ or *Chorioamnionitis/ or *Sepsis/ or *Fetal Membranes, Premature Rupture/ or *Pregnancy/ or *Fetal Diseases/ or *Humans/ or "fetal sepsis".mp. or *Pregnancy Complications, Infectious/ (124620)
6. "placenta".mp. or *Placenta/ or *Placenta Diseases/ (84976)
7. 1 and 2 and 3 and 4 and 5 and 6 (19)

                  **Total n=19**

Database: **Scopus**

<1960 to Present>

Search Strategy:

------------------------

acute villitis AND bacteria AND chorioamnionitis AND fetal intravascular organism* AND fetal sepsis AND placenta

**                   Total n=1**

Database: **Web of Science**

<1846 to Present>

Search Strategy:

------------------------

acute villitis AND bacteria AND chorioamnionitis AND fetal intravascular organism* AND fetal sepsis AND placenta

                   **Total n=2**

The initial electronic search yielded 19 results from Medline, two from Web of Science, one from Scopus and two from the citation search adding up to a total of 24 hits, one of which was excluded for duplication.^6^ The titles and abstracts of the remaining 23 studies were screened for inclusion, out of which 18 were removed for not meeting the inclusion criteria. Five papers were then fully read, and one further study was excluded for not reporting specifically on the presence of microorganisms in the fetal capillaries of the placenta.^17^ The final list included three case reports and a short case-series.^5,6,8^
